# Acute post-exercise blood pressure responses to sprint interval exercise in humans: a systematic review and structured synthesis

**DOI:** 10.3389/fphys.2026.1865794

**Published:** 2026-07-08

**Authors:** Lina Ge, Dandan Kong, Mingmei Chen, Shuting Xu, Chenzhe Ma, Ling Wang, Weibao Liang

**Affiliations:** 1 School of Physical Education, Xi'an University of Architecture and Technology, Xi’an, China; 2 Guangzhou Xinhua University, Guangzhou, China; 3 Zhanjiang University of Science and Technology, Zhanjiang, China; 4 The First Affiliated Hospital of Jinan University, Guangzhou, China; 5 Department of Physical Education, Kunsan National University, Gunsan-si, Republic of Korea; 6 Shijiazhuang Institute of Railway Technology, Shijiazhuang, China; 7 Faculty of Health Sciences and Sports, Macao Polytechnic University, Macao, Macao SAR, China

**Keywords:** acute exercise, blood pressure, crossover trial, descriptive quantitative synthesis, exercise physiology, post-exercise hypotension, sprint interval exercise, structured synthesis

## Abstract

**Background:**

Post-exercise hypotension is an acute systemic recovery response that links exercise physiology with blood pressure control, yet the response to all-out sprint interval exercise is less clearly defined than responses to moderate-intensity continuous or submaximal interval exercise.

**Methods:**

Six databases were searched from inception to 1 February 2026. Controlled acute human studies were eligible when they examined a single bout of sprint interval exercise (SIE) or a closely equivalent all-out, repeated-sprint, Wingate-based or supramaximal interval protocol and reported post-exercise blood pressure. Two reviewers independently screened records, extracted data and assessed risk of bias. Study-level mean differences were organized by comparator family and assessment window. Because exact passive-control data were sparse, crossover-aware exploratory quantitative summaries were retained only as descriptive aids, using an assumed within-participant correlation of 0.50 with sensitivity analyses. Values available only as figure-digitized means without extractable dispersions were retained for descriptive synthesis only.

**Results:**

Nine randomized crossover studies involving 136 participants were included. The exact passive/no-exercise control evidence was small: three studies with 31 participants contributed systolic blood pressure (SBP) data and two studies with 21 participants contributed diastolic blood pressure (DBP) data at the first extractable exact time point beyond 60 min. Study-level SBP mean differences were −3.0, −5.0 and −8.0 mmHg, indicating a directionally consistent short-term systolic reduction after SIE. Study-level DBP mean differences were −3.0 and −7.0 mmHg, providing only sparse preliminary evidence for DBP. In protocol-comparator studies, all three 45-min peripheral SBP estimates favored longer over shorter recovery intervals (−7.0, −7.5 and −5.0 mmHg). Exploratory quantitative summaries are reported in the full text and figures as descriptive aids only. Evidence for 24-h ambulatory blood pressure was limited to one study and did not establish a durable sprint-specific effect beyond the acute recovery window.

**Conclusion:**

Single-bout sprint interval exercise may elicit short-term post-exercise reductions in SBP and possibly DBP, but the evidence remains constrained by very small crossover study samples, heterogeneous protocols, incomplete paired-data reporting and reliance on assumed within-participant correlations. The findings support sprint interval exercise as an acute cardiovascular physiology stimulus, but they are not clinically definitive and do not establish superiority over other exercise modes. Registration: PROSPERO CRD420261366067; registered after screening had begun and before final quantitative synthesis.

**Systematic Review Registration:**

https://www.crd.york.ac.uk/PROSPERO/, identifier CRD420261366067.

## Graphical Abstract

**Figure:**
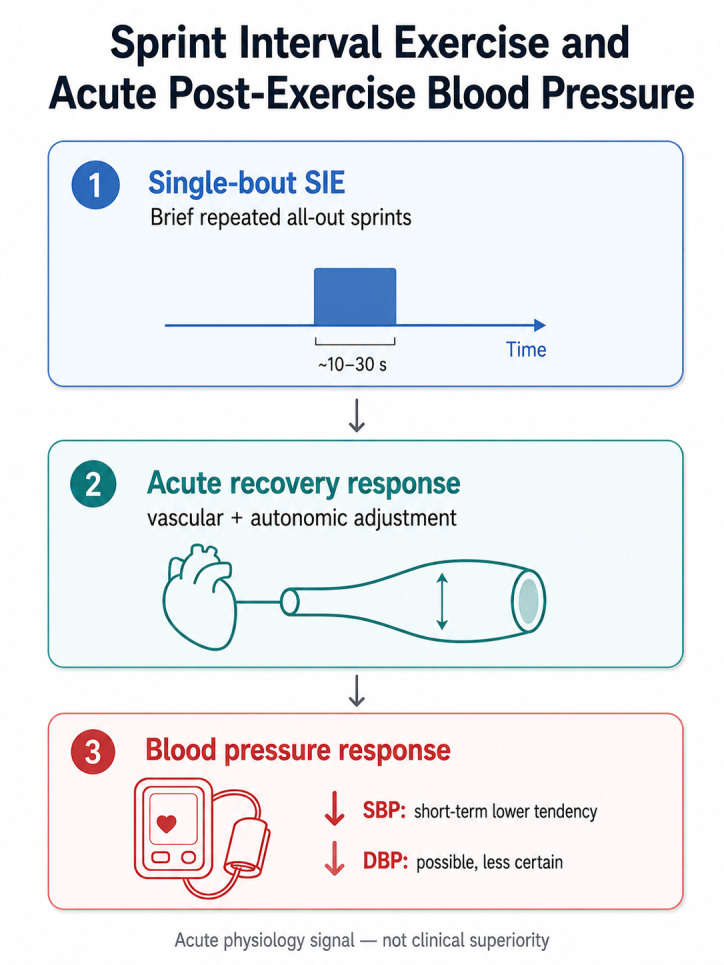
Schematic overview of the acute blood pressure response following a single bout of sprint interval exercise (SIE). Brief repeated all-out sprints initiate an acute recovery phase characterized by vascular and autonomic adjustment. During early post-exercise recovery, systolic blood pressure (SBP) may show a short-term lower tendency, whereas the diastolic blood pressure (DBP) response appears less consistent and more uncertain. This figure summarizes an acute physiological signal and should not be interpreted as evidence of sustained 24-hour blood pressure reduction or clinical superiority over other exercise modalities.

## Introduction

Post-exercise hypotension (PEH) describes the transient reduction in arterial blood pressure that occurs during recovery from a single bout of exercise. It is not a simple end-point artifact, but a recovery-state cardiovascular phenotype in which peripheral vascular conductance, cardiac output, autonomic regulation, baroreflex function and central blood-volume distribution interact after exercise cessation (Kenney and Seals, 1993; Halliwill, 2001; MacDonald, 2002; Halliwill et al., 2013; Romero et al., 2017). Because PEH can persist for minutes to hours, it is directly relevant to acute systemic and integrated multi-systemic exercise physiology.

PEH has translational relevance as a mechanistic acute response, but its clinical implications should be interpreted cautiously. Exercise recommendations and chronic training meta-analyses provide background that repeated exercise exposure can reduce resting blood pressure; however, these chronic treatment effects should not be inferred from a single acute PEH response (Pescatello et al., 2004; Cornelissen and Smart, 2013; Pescatello et al., 2015; Edwards et al., 2023). Acute PEH may be one mechanistic pathway through which repeated exercise contributes to blood-pressure adaptation, but a transient post-exercise response is not equivalent to a chronic treatment effect. Its magnitude and interpretation depend on participant characteristics, exercise mode, dose, intensity, timing of measurement and the comparator condition (Brito et al., 2018, 2019; Day et al., 2022).

Sprint interval exercise (SIE) is physiologically distinct from moderate-intensity continuous exercise and from submaximal high-intensity interval exercise. Typical SIE protocols use brief all-out or supramaximal bouts separated by recovery periods and therefore impose large but short-lived disturbances in power output, oxygen demand, local metabolite accumulation, vascular shear stress and autonomic activation (Burgomaster et al., 2005; Gibala et al., 2012). These features may amplify signals that promote sustained vasodilation after exercise, yet they may also produce transient pressor responses, symptoms during recovery or slower autonomic normalization in some individuals.

The primary literature on SIE and PEH is small and methodologically heterogeneous. Studies vary in participant age and blood-pressure status, sprint number and duration, recovery interval, exercise modality, comparator type, blood-pressure device, posture and assessment window. Some trials compare SIE with passive rest or no-exercise control, whereas others compare alternative SIE protocols or active exercise modalities. Combining all comparator structures into a single estimate would therefore obscure physiology and reduce interpretability.

The objective of this systematic review was to provide a structured synthesis of acute post-exercise blood-pressure responses to single-bout SIE in humans and to evaluate whether protocol structure, particularly recovery interval length, modifies the response. Where minimally comparable exact data were available, we also reported exploratory crossover-aware quantitative summaries to aid interpretation, without treating these estimates as confirmatory evidence. We expected the passive-control SBP evidence to be directionally consistent but limited, and we expected DBP, active-comparator and 24-h ambulatory evidence to be less certain because of sparse data and heterogeneous assessment windows.

## Methods

### Protocol, reporting and article type

This review was conducted according to the Preferred Reporting Items for Systematic Reviews and Meta-Analyses 2020 statement (Page et al., 2021). The present review was registered in PROSPERO (CRD420261366067) after screening had begun and before final quantitative synthesis; therefore, registration was not prospective relative to the start of screening, and this timing is acknowledged in the limitations. Registration was not prospective relative to the start of screening; this timing is acknowledged in the limitations. The manuscript is formatted as a Frontiers Systematic Review because it uses systematic methods to identify, categorize, analyze and report aggregated evidence from previous research. The review question was defined by population, intervention, comparator, outcomes and study design (PICOS). All extracted outcome data, effect-size inputs and figure source data are provided in **Supplementary File 2**.

### Eligibility criteria

Human acute experimental studies were eligible when they examined a single session of SIE or a closely equivalent repeated all-out, repeated-sprint, Wingate-based or supramaximal interval protocol and reported post-exercise blood pressure. Eligible populations included participants of any sex, age and health status. Eligible comparators included passive rest, no-exercise control, steady-state or continuous exercise, aerobic interval exercise, high-intensity interval exercise, endurance exercise or an alternative SIE protocol. Eligible outcomes were brachial or peripheral systolic blood pressure (SBP), diastolic blood pressure (DBP), mean arterial pressure (MAP), central blood pressure, ambulatory blood pressure and study-defined PEH area-under-the-curve measures.

Outcomes were organized for structured synthesis by assessment window as immediate recovery, 10–60 min, beyond 60 min and 24-h ambulatory blood pressure. The beyond-60-min exact passive-control window was a pragmatic data-availability window rather than an assumption that PEH peaks after 60 min; earlier 10–60-min evidence was summarized descriptively when exact paired or dispersion data were not extractable. Chronic training studies without extractable single-bout blood-pressure responses, animal studies, pure resistance or isometric interventions, interval protocols without all-out or supramaximal sprint characteristics, non-controlled reports and studies without extractable post-exercise blood-pressure data were excluded.

### Information sources and search strategy

Six databases were searched from inception to 1 February 2026: PubMed/MEDLINE, Web of Science Core Collection, Cochrane Library, Scopus, SPORTDiscus and Embase. The search combined terms for sprint interval training or exercise, repeated sprint exercise, Wingate protocols, all-out or supramaximal exercise, blood-pressure outcomes and post-exercise recovery. Human filters were used only where explicitly included in the supplied database strategy. The strategy was intentionally broad and included high-intensity interval training and sprint interval training indexing terms to maximize sensitivity; eligibility screening then excluded protocols that did not satisfy the acute SIE definition. Full query strings and database yields are provided in **Supplementary Table 1**.

### Study selection and data collection

Two reviewers (L.G. and D.K.) independently screened titles and abstracts. Potentially eligible reports were assessed in full text by the same reviewers. Disagreements were resolved by discussion, with W.L. adjudicating unresolved decisions. The final inclusion set was agreed before quantitative synthesis.

Two reviewers (L.G. and M.C.) independently extracted data using a piloted extraction workbook. Extracted items included design, country, participant characteristics, blood-pressure status, sample size, intervention and comparator protocols, sprint number and duration, recovery interval, exercise mode, blood-pressure device and posture, assessment times, adverse events, numerical outcomes, dispersion measures and statistical model information. D.K. verified numerical entries against source reports, and W.L. adjudicated unresolved discrepancies. Exact tables were prioritized over text values; text values were prioritized over published figure-derived values. When only published figures were available, means were digitized for descriptive time-course synthesis, but estimates without extractable dispersions were not included in weighted quantitative summaries. Values reported as standard errors were converted to standard deviations when sample size was unambiguous. Outcome values were not imputed when neither exact values nor defensible derivations were available.

### Risk of bias and certainty of evidence

Two reviewers (D.K. and S.X.) independently assessed included studies with the Cochrane Risk of Bias 2 framework for randomized trials, with crossover-specific attention to allocation order, period or carryover effects, deviations from intended interventions, missing outcome data, outcome measurement and selective reporting (Sterne et al., 2019). Disagreements were resolved by consensus and adjudicated by W.L. when required.

Certainty of evidence for each structured synthesis family was evaluated using GRADE principles, considering risk of bias, inconsistency, indirectness, imprecision and publication bias (Guyatt et al., 2008; Higgins et al., 2024). Review-level contextual sources used in the background and discussion were checked for methodological credibility and overlap safeguards with AMSTAR-2 and corrected covered area principles; no review-level pooled estimate contributed data to the present primary-study synthesis (Shea et al., 2017; Pieper et al., 2014; Hennessy and Johnson, 2020).

### Effect measures and synthesis strategy

The primary synthesis family compared SIE with passive rest or no-exercise control. The primary descriptive effect measure was the study-level mean difference in SBP or DBP, calculated as intervention minus comparator; negative values therefore favor lower blood pressure after SIE. For the passive-control exact-data summary—not as a review-level eligibility criterion—the first extractable exact post-exercise time point beyond 60 min was used as a pragmatic data-availability window because exact 10–60-min values with the dispersion and/or paired information needed for weighted exploratory summaries were incompletely reported across studies. A sensitivity analysis used the latest available exact time point beyond 60 min. A second synthesis family compared longer versus shorter recovery intervals between sprint bouts in studies that directly manipulated SIE protocol structure; these 45-min Ketelhut estimates were analyzed separately and were not included in the passive-control beyond-60-min family. Active-exercise comparator studies and outcomes available only as figure-digitized means without extractable dispersions were summarized descriptively rather than combined into a pooled estimate.

For crossover studies, standard errors of paired mean differences were calculated from group standard deviations using an assumed within-participant correlation of 0.50, consistent with standard approaches when paired standard deviations are unavailable (Elbourne et al., 2002; Higgins et al., 2024). The paired standard error was calculated as square root of [(SD_intervention² + SD_comparator² − 2r × SD_intervention × SD_comparator)/n]. Sensitivity analyses repeated the SBP and DBP summaries across assumed correlations from 0.00 to 0.90 and evaluated the effect of time-window choice. Exploratory inverse-variance random-effects summaries were used only to supplement the structured study-level synthesis because protocol and population heterogeneity were expected *a priori*. Heterogeneity was summarized with I² and τ² but was not used for strong inference because synthesis families contained only two or three studies/estimates. Funnel plots were considered exploratory only because no quantitative family included at least ten studies. All model-derived confidence intervals and heterogeneity statistics were interpreted as estimation-based exploratory summaries rather than definitive confirmatory evidence. Effect-size inputs and formula-based audit calculations were documented in the spreadsheet-based extraction/audit workbook; all reported exploratory quantitative summaries and assumed-correlation sensitivity sweeps are reproduced in **Supplementary File 3** using R and the metafor package, with model specifications stated in the script (Viechtbauer, 2010).

## Results

### Study selection

**Figure 1:**
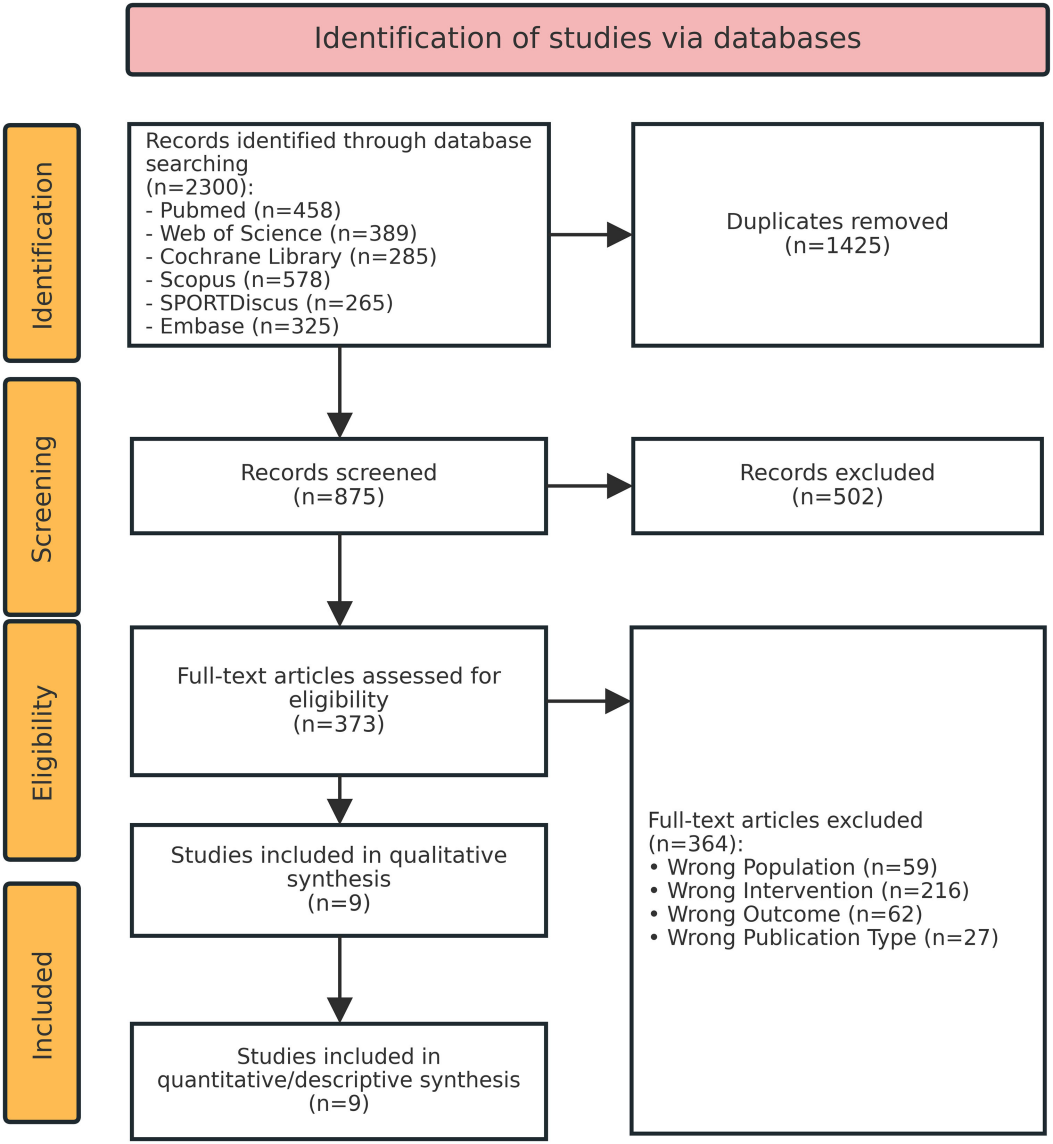
PRISMA 2020 flow diagram for study identification, screening, eligibility assessment and inclusion.

The database search identified 2,300 records. After removal of 1,425 duplicate records, 875 records were screened by title and abstract; 502 were excluded and 373 full-text reports were assessed for eligibility. A total of 364 full-text reports were excluded, most commonly because the intervention was not an eligible SIE protocol, the population was outside the review scope, the outcome did not provide post-exercise blood-pressure data or the study design was ineligible. Nine studies were included in the qualitative synthesis and contributed to at least one structured, quantitative or descriptive synthesis family (Stuckey et al., 2012; Burns et al., 2012; Chan and Burns, 2013; Angadi et al., 2015; Graham et al., 2016; Jones et al., 2021; Ketelhut et al., 2023a, 2023; McCarthy et al., 2023). The PRISMA flow diagram is shown in **Figure 1**.

### Study characteristics

The nine included studies enrolled 136 participants and used randomized crossover acute experimental designs. Most participants were healthy, physically active or normotensive; one study enrolled middle-aged adults with mixed normotensive, prehypertensive and hypertensive status. Protocols ranged from two to six 30-s Wingate-style bouts, four 30-s all-out bouts with manipulated recovery intervals, eight 15-s all-out treadmill sprints, 30 repeated 8-s sprints and single-versus-multiple Wingate comparisons. Comparator families included passive or no-exercise control, active aerobic exercise comparators and alternative SIE protocols. Study and protocol characteristics are summarized in **Table 1**.

**Table 1:** Study and protocol characteristics of included acute sprint interval exercise studies.

Study	Participants and design	Sprint interval exercise exposure	Comparator(s) and BP window	Synthesis role
Stuckey et al. (2012)	9 healthy recreationally active men; randomized crossover acute experiment	4 × 30-s Wingate bouts with 4-min active recovery	1 × 30-s Wingate; SBP/DBP and autonomic outcomes to 120 min	Narrative/mechanistic; comparator not passive control
Burns et al. (2012)	10 healthy adolescents; random balanced crossover	2 × 30-s Wingate bouts with 4-min recovery	Rest control; seated SBP/DBP to 90 min	Passive-control study-level SBP summary; exploratory SBP summary
Chan and Burns (2013)	10 healthy men; random balanced crossover	4 × 30-s sprint bouts with 4.5-min recovery	Rest control; seated SBP/DBP to 120 min	Passive-control study-level SBP and DBP summary; exploratory SBP/DBP summary
Angadi et al. (2015)	11 healthy young adults completing SIE; randomized crossover with 4 conditions	6 × 30-s Wingate bouts with 4-min active recovery	Control, steady-state exercise and aerobic interval exercise; seated SBP/DBP every 15 min for 3 h	Passive-control study-level SBP and DBP summary; exploratory SBP/DBP summary; active-comparator narrative
Graham et al. (2016)	12 untrained men; randomized counterbalanced crossover	5 × 30-s maximal leg or arm intervals with 4.5-min recovery	50-min endurance exercise; MAP and PEH area-under-the-curve, including longer follow-up	Active-comparator narrative due to outcome metric
Jones et al. (2021)	16 normotensive adults; randomized crossover	30 × 8-s maximal sprints with 32-s recovery	HIIE and MICE; seated SBP/DBP at 5–90 min	Active-comparator descriptive synthesis
Ketelhut et al. (2023b)	30 healthy trained adults; randomized crossover	4 × 30-s all-out bouts comparing 3-min vs 1-min recovery	Longer-vs-shorter SIE recovery protocol; peripheral and central BP to 45 min	Protocol-comparator study-level and exploratory summary
Ketelhut et al. (2023a)	24 matched trained adults (younger and older subgroups); randomized crossover	4 × 30-s all-out bouts comparing 3-min vs 1-min recovery	Longer-vs-shorter SIE recovery protocol by age subgroup; peripheral and central BP to 45 min	Protocol-comparator study-level and exploratory summary
McCarthy et al. (2023)	14 top-aged adults; randomized crossover; 13 with valid 24-h ABP	8 × 15-s all-out treadmill sprints with 2-min passive rest	Control, MICT and HIIT; laboratory BP to 120 min and 24-h ABP	Active-comparator and 24-h descriptive synthesis

### Risk of bias

**Figure 2:**
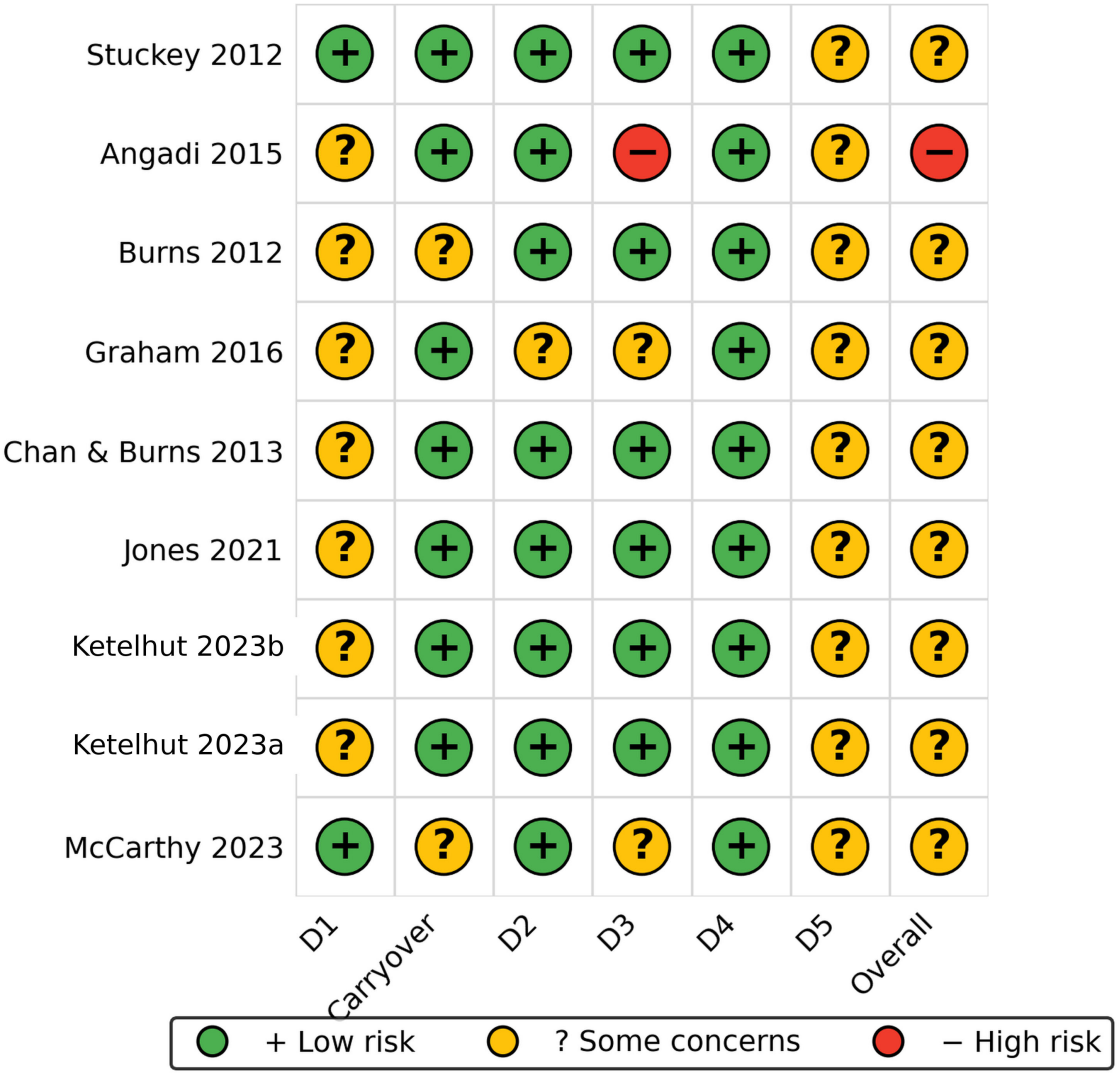
Risk-of-bias traffic-light plot for the nine included randomized crossover studies assessed with the Cochrane RoB 2 framework. Symbols and colors indicate judgment categories: plus sign, low risk; question mark, some concerns; minus sign, high risk.

Risk-of-bias judgments are summarized in **Figure 2** and detailed in **Supplementary Table 4**. One study was judged at high overall risk of bias because missing outcome data were plausibly related to the sprint intervention (Angadi et al., 2015). The remaining studies were generally judged as having some concerns, mainly because of incomplete reporting of randomization procedures, limited information about period or carryover control, small sample sizes and incomplete reporting of paired outcome statistics. No study was excluded from synthesis solely on the basis of risk of bias.

### Passive or no-exercise control synthesis

**Figure 3:**
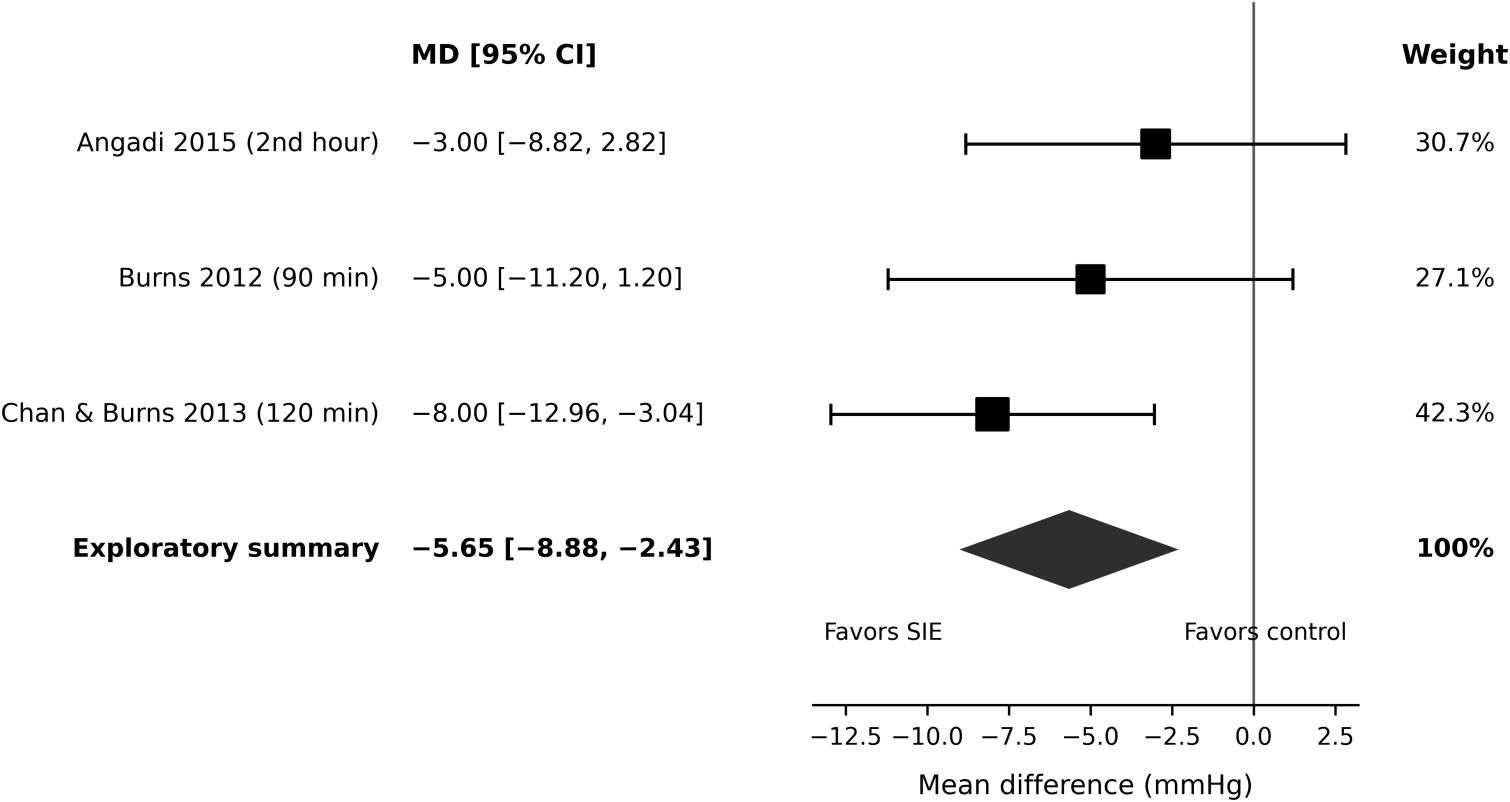
Exploratory forest plot for systolic blood pressure at the first extractable exact post-exercise time point beyond 60 min in the passive or no-exercise control synthesis. Mean differences are intervention minus comparator; negative values favor lower blood pressure after sprint interval exercise. The summary diamond is retained as a descriptive quantitative aid because only three studies contributed data.

**Figure 4:**
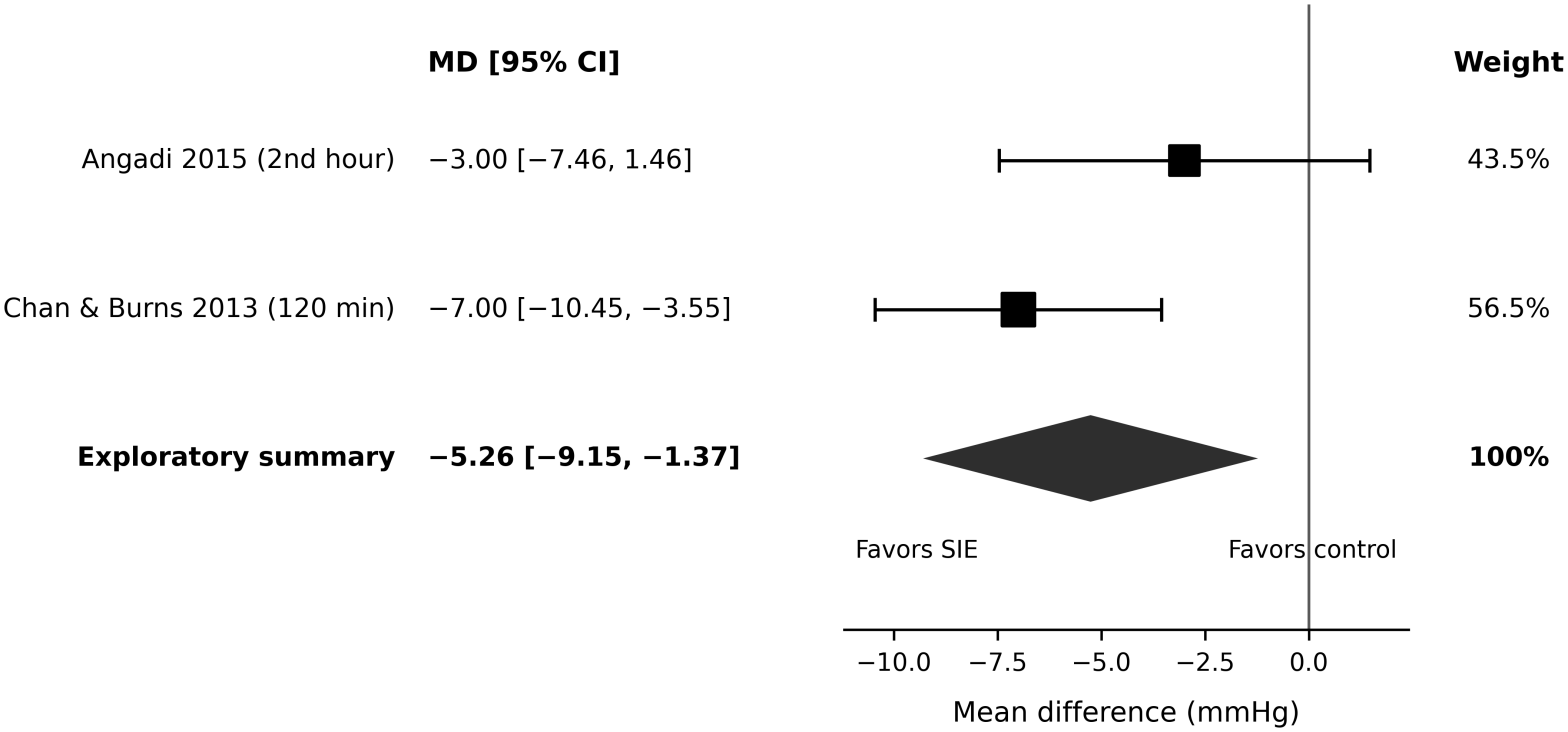
Exploratory forest plot for diastolic blood pressure at the first extractable exact post-exercise time point beyond 60 min in the passive or no-exercise control synthesis. Mean differences are intervention minus comparator; negative values favor lower blood pressure after sprint interval exercise. The summary diamond is retained as a descriptive quantitative aid because only two studies contributed data.

Three studies contributed exact SBP data to the passive or no-exercise control synthesis at the first extractable exact time point beyond 60 min. The study-level mean differences were directionally consistent but came from small crossover samples: −3.0 mmHg in Angadi et al. (2015; second-hour mean), −5.0 mmHg in Burns et al. (2012; 90 min) and −8.0 mmHg in Chan and Burns (2013; 120 min). The exploratory random-effects summary was −5.65 mmHg (95% CI −8.88 to −2.43; I² = 0%; **Figure 3**; **Table 2**). Two studies contributed exact DBP data, providing only sparse preliminary evidence. Study-level mean differences were −3.0 mmHg in Angadi et al. (2015; second-hour mean) and −7.0 mmHg in Chan and Burns (2013; 120 min), with an exploratory summary of −5.26 mmHg (95% CI −9.15 to −1.37; I² = 48%; **Figure 4**; **Table 2**). Because k was two to three, these exploratory summary values should be read only as descriptive quantitative summaries of compatible exact data, not as precise or confirmatory estimates.

**Table 2:** Summary of quantitative synthesis families and main estimates.

Synthesis family	Outcome/time window	Study-level evidence	Study-specific mean differences	Exploratory summary/certainty	Interpretation
Passive or no-exercise control	SBP, first extractable exact time point >60 min	3 studies; 31 participants	Angadi et al., 2015: −3.0 mmHg (95% CI −8.8 to 2.8); Burns et al., 2012: −5.0 (−11.2 to 1.2); Chan and Burns, 2013: −8.0 (−13.0 to −3.0)	MD −5.65 mmHg (95% CI −8.88 to −2.43); I² = 0%; low certainty	Directionally consistent short-term systolic reduction; exploratory summary only
Passive or no-exercise control	DBP, first extractable exact time point >60 min	2 studies; 21 participants	Angadi et al., 2015: −3.0 mmHg (95% CI −7.5 to 1.5); Chan and Burns, 2013: −7.0 (−10.5 to −3.6)	MD −5.26 mmHg (95% CI −9.15 to −1.37); I² = 48%; very low certainty	Possible diastolic reduction; sparse and preliminary
Passive-control sensitivity	SBP, latest exact time point >60 min	3 studies; 31 participants	Angadi et al., 2015 third-hour mean; Burns et al., 2012–90 min; Chan and Burns, 2013–120 min	MD −4.76 mmHg (95% CI −8.96 to −0.57); I² = 42.75%; sensitivity analysis	Direction retained with smaller magnitude
Passive-control sensitivity	DBP, latest exact time point >60 min	2 studies; 21 participants	Angadi et al., 2015 third-hour mean; Chan and Burns, 2013–120 min	MD −3.65 mmHg (95% CI −10.50 to 3.21); I² = 83.10%; sensitivity analysis	Uncertain DBP response at latest window
Protocol comparator	Peripheral SBP at 45 min; longer vs shorter recovery	3 estimates; 54 participants	Ketelhut et al., 2023b: −7.0 mmHg (95% CI −10.5 to −3.5); Ketelhut et al., 2023a older: −7.5 (−13.9 to −1.1); Ketelhut et al., 2023a younger: −5.0 (−11.0 to 1.0)	MD −6.67 mmHg (95% CI −9.41 to −3.92); I² = 0%; low certainty	Lower pSBP after longer-recovery protocols; limited to closely related studies
Protocol comparator	Peripheral DBP at 45 min; longer vs shorter recovery	3 estimates; 54 participants	Ketelhut et al., 2023b: −1.0 mmHg (95% CI −4.1 to 2.1); Ketelhut et al., 2023a older: −1.0 (−4.7 to 2.7); Ketelhut et al., 2023a younger: +1.0 (−4.4 to 6.4)	MD −0.68 mmHg (95% CI −2.84 to 1.48); I² = 0%; low certainty	No clear pDBP difference
Single-study descriptive evidence	24-h ambulatory SBP/DBP after SIE vs control	1 study; 13 participants with valid ABP	McCarthy et al., 2023: SBP 121 ± 12 vs 123 ± 13 mmHg; DBP 75 ± 8 vs 75 ± 10 mmHg	No pooled model; very low certainty	No established durable 24-h SIE-specific effect

### Protocol-comparator synthesis

**Figure 5:**
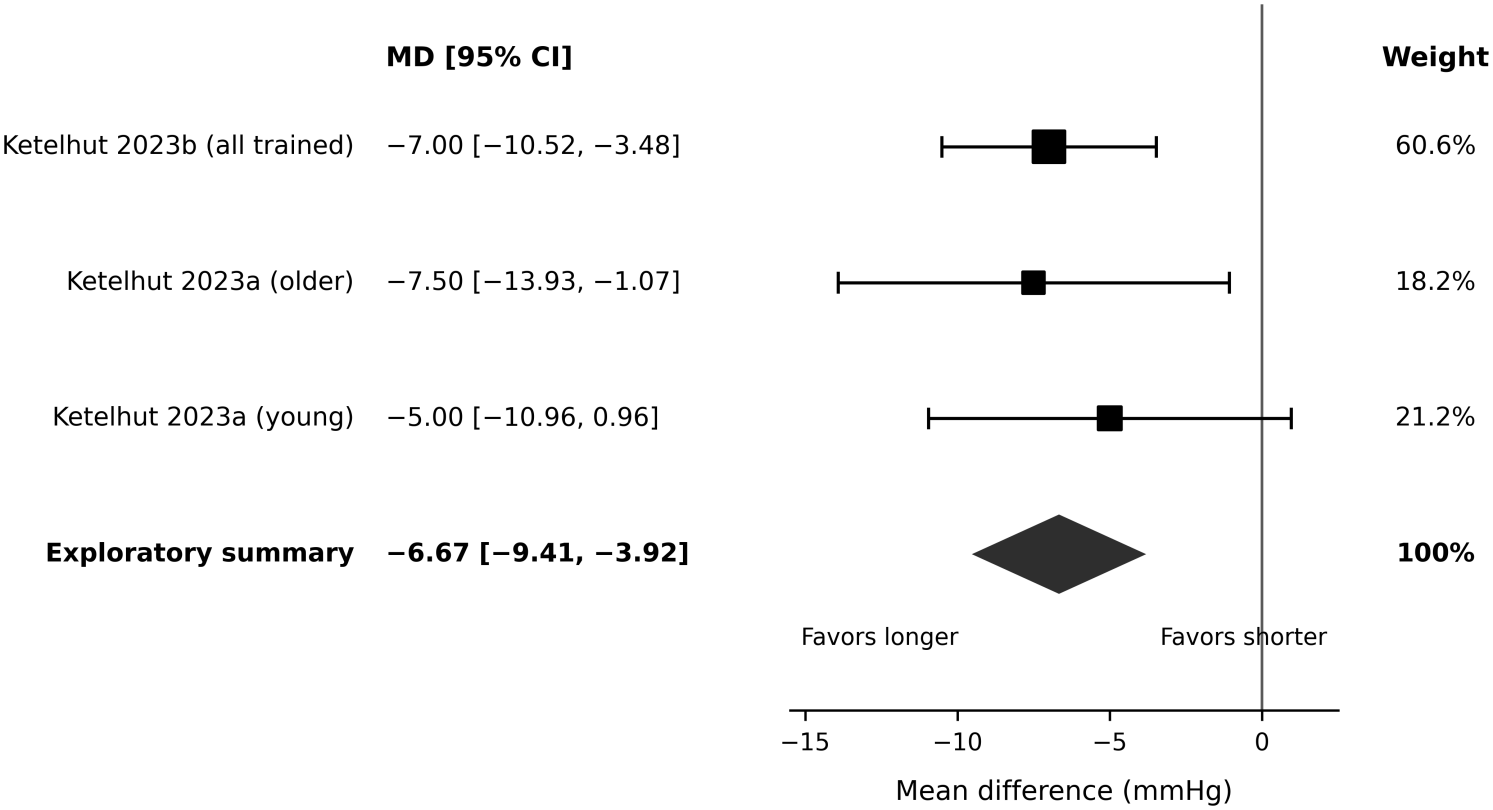
Exploratory forest plot for peripheral systolic blood pressure at 45 min comparing longer versus shorter recovery intervals between sprint bouts. Negative values favor the longer-recovery sprint interval exercise protocol.

Three 45-min estimates from two Ketelhut protocol-comparison studies compared longer versus shorter recovery intervals between sprint bouts; these estimates were handled as a separate protocol-comparator family and were not included in the passive/no-exercise control beyond-60-min exact-data summary. All three peripheral SBP estimates favored longer recovery (Ketelhut et al., 2023b: −7.0 mmHg; Ketelhut et al., 2023a older subgroup: −7.5 mmHg; Ketelhut et al., 2023a younger subgroup: −5.0 mmHg). The exploratory random-effects summary was −6.67 mmHg (95% CI −9.41 to −3.92; I² = 0%; **Figure 5**; **Table 2**). The corresponding peripheral DBP estimates were small and inconsistent in direction (−1.0, −1.0 and +1.0 mmHg; exploratory summary −0.68 mmHg, 95% CI −2.84 to 1.48; I² = 0%; **Table 2**; **Supplementary Figure 2**). These findings suggest that recovery-interval manipulation may influence the systolic component of the PEH response, but the evidence base is limited to closely related protocol-comparison studies and cannot define an optimal sprint dose.

### Sensitivity analyses

**Figure 6:**
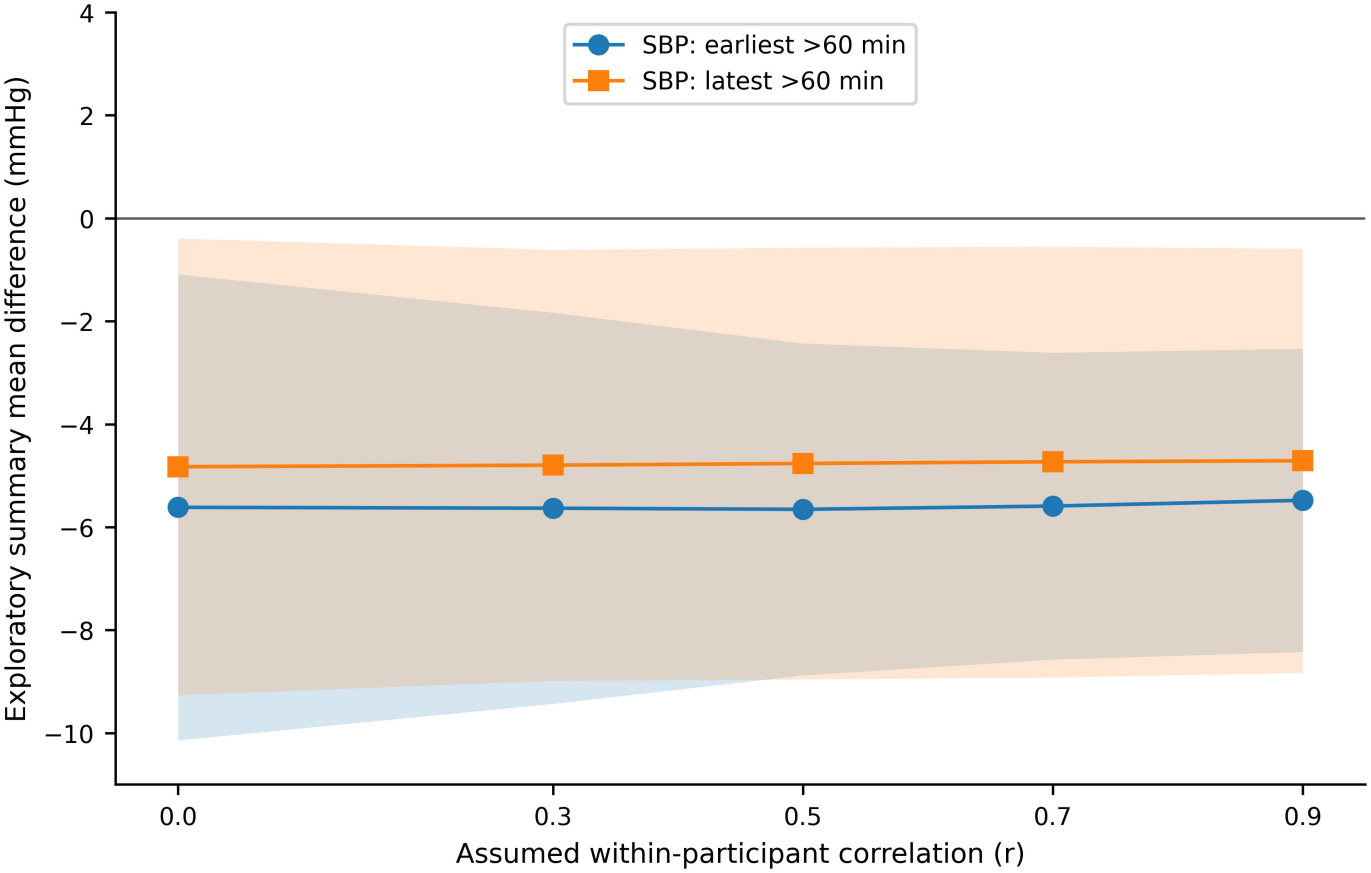
Sensitivity analysis for the passive-control systolic blood pressure summary across assumed within-participant correlations and alternative beyond-60-min exact-data choices. Negative values favor lower blood pressure after sprint interval exercise.

Time-window sensitivity analyses showed that the SBP summary remained directionally consistent when the latest available time point beyond 60 min was used, although the magnitude was smaller (MD −4.76 mmHg, 95% CI −8.96 to −0.57; I² = 42.75%; **Table 2**). Correlation-sweep analyses indicated that the direction of the SBP summary was not dependent on a single assumed within-participant correlation, but the width of confidence intervals and the heterogeneity estimates changed across assumptions (**Figure 6**; **Supplementary Table 5**). DBP sensitivity estimates were less stable, especially when the latest time window was used (MD −3.65 mmHg, 95% CI −10.50 to 3.21; I² = 83.10%; **Table 2**), consistent with a smaller evidence base and greater uncertainty.

### Active comparators, ambulatory blood pressure and descriptive time-course evidence

Studies comparing SIE with active exercise modalities were not pooled as a single effect because comparators, outcome metrics and measurement schedules differed substantially. Angadi et al. (2015) compared SIE with steady-state and aerobic interval exercise; Graham et al. (2016) emphasized MAP and PEH area-under-the-curve responses after limb-specific interval and endurance exercise; Jones et al. (2021) compared SIE with high-intensity interval and moderate continuous exercise; and McCarthy et al. (2023) compared SIE with MICT, HIIT and no-exercise control. Published figure-derived acute time-course means were retained in **Supplementary File 2** for transparency, but were not included in any weighted quantitative summary when exact dispersions were unavailable. Evidence for 24-h ambulatory blood pressure was limited to McCarthy et al. (2023): 24-h average SBP was 121 ± 12 mmHg after SIE and 123 ± 13 mmHg after control, whereas DBP was 75 ± 8 and 75 ± 10 mmHg, respectively. This single-study evidence should be considered preliminary and did not establish a durable 24-h SIE-specific blood-pressure reduction beyond the acute recovery period.

## Discussion

This systematic review with structured synthesis suggests that a single bout of SIE may elicit a short-term reduction in post-exercise SBP compared with passive or no-exercise control. The three exact passive-control studies were directionally consistent, with study-level SBP mean differences ranging from −3.0 to −8.0 mmHg; the corresponding exploratory summary was approximately −6 mmHg. DBP was also lower in the limited exact evidence base, but this inference rests on only two studies and is more uncertain. Protocol-comparator evidence further suggested that longer recovery intervals between sprint bouts may be associated with lower 45-min peripheral SBP than shorter intervals. These findings should be interpreted as physiologically plausible, hypothesis-generating evidence from small crossover trials, not as definitive clinical treatment effects.

The SBP response is consistent with established PEH physiology. After dynamic exercise, arterial pressure can fall because sustained peripheral vasodilation is not fully offset by cardiac output. Mechanistic contributors include altered sympathetic outflow, attenuated sympathetic vasoconstrictor responsiveness, histamine-mediated vasodilation, nitric oxide and prostaglandin pathways in some settings, baroreflex resetting and redistribution of central blood volume (Halliwill, 2001; Halliwill et al., 2013; Romero et al., 2017). SIE may intensify several of these stimuli despite low total exercise volume because all-out sprints create high local metabolic demand, large fluctuations in muscle perfusion and marked autonomic activation (Burgomaster et al., 2005; Gibala et al., 2012). The available studies do not directly identify the dominant pathway, but the response pattern is biologically plausible within current PEH models.

The observed effect of sprint recovery interval length is also physiologically plausible. Longer between-sprint recovery may allow higher-quality repeated sprints, greater cumulative mechanical work and a larger vascular stimulus while permitting partial restoration of perfusion pressure before subsequent bouts. Shorter recovery intervals may reduce external power output or shift the recovery balance among cardiac output, stroke volume, heart rate and vascular conductance. Importantly, these interpretations remain hypotheses. The present evidence indicates that protocol structure should not be treated as interchangeable when post-exercise blood pressure is the outcome, but it does not define the optimal sprint dose for health or performance.

The findings should not be read as evidence that SIE is superior to moderate continuous or submaximal interval exercise for acute blood-pressure reduction. Active-comparator studies produced heterogeneous patterns, with some suggesting similar PEH magnitude across exercise modes and others suggesting different time courses or larger responses for aerobic interval or endurance exercise at specific windows. This is compatible with broader PEH literature showing that participant blood-pressure status, age, body composition, exercise duration, intensity, posture, device and assessment timing can alter both the magnitude and interpretation of acute blood-pressure responses (Brito et al., 2018, 2019; Day et al., 2022). SIE should therefore be considered one possible acute cardiovascular stimulus rather than a universally stronger antihypertensive exercise option.

The clinical relevance of a transient 5–6 mmHg SBP reduction depends on context and should not be equated with chronic resting blood-pressure reductions from exercise training trials. Chronic reductions of similar magnitude in resting SBP are meaningful at the population level, but this comparison is provided only to contextualize numerical scale and cannot be used to infer chronic benefit from a single SIE bout; repeated acute reductions have been proposed as one possible pathway linking exercise exposure with longer-term blood-pressure adaptation (Cornelissen and Smart, 2013; Pescatello et al., 2015; Wegmann et al., 2018; Edwards et al., 2023). However, most included SIE studies enrolled young, healthy or normotensive participants. All-out exercise may be less tolerable in sedentary, hypertensive or cardiometabolic-risk populations, and acute post-exercise symptoms or adverse events require careful reporting. The present evidence supports physiological plausibility under controlled laboratory conditions, but it does not justify broad clinical prescription without population-specific safety, feasibility and comparative-effectiveness trials.

This review also identifies reporting practices that limit reproducibility. Crossover trials often reported condition means and standard deviations but not within-participant standard deviations, within-participant correlations, period effects, sequence allocation, washout adequacy or individual response distributions. As a result, exploratory quantitative summaries required sensitivity analyses based on assumed correlations, and some otherwise relevant time-course data could not be included in weighted models. Future studies should report baseline-adjusted and control-adjusted changes, paired standard deviations, exact time-point means and dispersions, randomization sequence generation, allocation order, washout duration, missing-data reasons, adverse events, protocol fidelity and device calibration procedures. Ambulatory blood-pressure monitoring and prespecified laboratory assessment windows would also strengthen inference about the duration of SIE-related PEH.

Strengths of this review include a broad multi-database search, explicit separation of passive-control, active-comparator and protocol-comparator families, duplicate screening and extraction, crossover-aware effect-size handling, RoB 2 assessment, GRADE-based certainty judgments and transparent release of extraction and analysis inputs. These choices reduce the risk of overgeneralizing across incompatible comparator structures and make the statistical assumptions auditable.

Several limitations should temper interpretation. Each quantitative summary contained few studies or estimates and small total sample sizes, so τ² and I² have low inferential value and the exploratory summary estimates are sensitive to individual studies. The beyond-60-min passive-control exact-data window reflected incomplete reporting of extracTable 10–60-min values and paired/dispersion information in the primary studies; it should not be interpreted as a predefined physiological peak window or as a claim that PEH peaks after 60 min, and 10–60-min evidence remains important for future trials. Most participants were healthy or normotensive, limiting inference for hypertension management. SIE protocols differed in sprint number, duration, recovery interval, modality and posture, and these differences are physiologically meaningful. Some outcomes were available only as published figures or non-standard PEH area-under-the-curve metrics; these data were retained for descriptive interpretation or excluded from weighted exploratory summaries when exact dispersions were unavailable. Within-participant correlations and paired standard deviations were rarely reported, so the weighted exploratory summaries required assumed-correlation variance estimation; sensitivity sweeps assessed robustness across plausible assumptions but cannot remove this source of uncertainty. Funnel plots were exploratory because the number of studies was below accepted thresholds for formal small-study-effect assessment. The review was registered after screening had begun rather than prospectively before screening, which is an additional reporting limitation. Finally, the review was restricted by information available in published reports and **Supplementary Materials**; no unpublished outcome data were used for weighted exploratory summaries.

Future trials should prioritize mechanistically informative and reproducible designs. In adults with elevated blood pressure, modified or lower-barrier sprint-interval formats should be compared with both passive controls and clinically relevant active exercise comparators. Trials should integrate laboratory blood pressure, central hemodynamics, autonomic indices, vascular conductance, symptoms and 24-h ambulatory monitoring. Such designs would help determine whether the acute systolic response observed here reflects a robust SIE phenotype, a protocol-specific response or a finding mainly applicable to young healthy samples.

## Conclusion

Single-bout sprint interval exercise may produce short-term post-exercise reductions in systolic blood pressure compared with passive or no-exercise control, and limited evidence suggests a possible concurrent diastolic reduction. Longer recovery intervals between sprint bouts may be associated with lower 45-min peripheral systolic blood pressure than shorter intervals. The current evidence supports SIE as a physiologically plausible acute cardiovascular stimulus, but it remains too small and heterogeneous to establish clinical superiority over other exercise modes, durable 24-h blood-pressure effects or definitive clinical efficacy.

## Data Availability

The original contributions presented in the study are included in the article/**Supplementary Material**. Further inquiries can be directed to the corresponding authors.
