# Supplementary material for: Acute post-exercise blood pressure responses to sprint interval exercise in humans: a systematic review and structured synthesis
Source: Front Physiol. 2026 Jul 8;17:1865794. doi: 10.3389/fphys.2026.1865794 (PMC13388036; doi:10.3389/fphys.2026.1865794)

**Supplementary Appendix**

# Supplementary Table S1. Search strategy and database yields.

| **Database** | **Search date** | **Records identified** | **Exact query strings** |
| --- | --- | --- | --- |
| PubMed/MEDLINE | 2026-02-01 | 458 | ("High-Intensity Interval Training"[Mesh] OR "sprint interval training"[Title/Abstract] OR "sprint interval exercise"[Title/Abstract] OR "sprint interval"[Title/Abstract] OR "repeated sprint"[Title/Abstract] OR "repeated-sprint"[Title/Abstract] OR "Wingate"[Title/Abstract] OR "all-out"[Title/Abstract] OR "supramaximal exercise"[Title/Abstract] OR "supramaximal interval exercise"[Title/Abstract] OR "SIT"[Title/Abstract] OR "SIE"[Title/Abstract]) AND ("Blood Pressure"[Mesh] OR "Blood Pressure Monitoring, Ambulatory"[Mesh] OR "blood pressure"[Title/Abstract] OR "systolic blood pressure"[Title/Abstract] OR "diastolic blood pressure"[Title/Abstract] OR "mean arterial pressure"[Title/Abstract] OR "postexercise hypotension"[Title/Abstract] OR "post-exercise hypotension"[Title/Abstract] OR "PEH"[Title/Abstract] OR "SBP"[Title/Abstract] OR "DBP"[Title/Abstract] OR "MAP"[Title/Abstract]) AND ("Post-Exercise Recovery"[Mesh] OR "postexercise"[Title/Abstract] OR "post exercise"[Title/Abstract] OR "post-exercise"[Title/Abstract] OR "after exercise"[Title/Abstract] OR "following exercise"[Title/Abstract] OR "recovery"[Title/Abstract] OR "acute"[Title/Abstract]) NOT (animals[Mesh] NOT humans[Mesh]) |
| Web of Science | 2026-02-01 | 389 | TS=(("sprint interval training" OR "sprint interval exercise" OR "sprint interval" OR "repeated sprint" OR "repeated-sprint" OR Wingate OR "all-out" OR "supramaximal exercise" OR "supramaximal interval exercise" OR SIT OR SIE) AND ("blood pressure" OR "systolic blood pressure" OR "diastolic blood pressure" OR "mean arterial pressure" OR "postexercise hypotension" OR "post-exercise hypotension" OR PEH OR SBP OR DBP OR MAP) AND (postexercise OR "post exercise" OR "post-exercise" OR "after exercise" OR "following exercise" OR recovery OR acute)) |
| Cochrane Library | 2026-02-01 | 285 | #1 ("sprint interval training" OR "sprint interval exercise" OR "sprint interval" OR "repeated sprint" OR "repeated-sprint" OR Wingate OR "all-out" OR "supramaximal exercise" OR "supramaximal interval exercise" OR SIT OR SIE),ab,kw  #2 ("blood pressure" OR "systolic blood pressure" OR "diastolic blood pressure" OR "mean arterial pressure" OR "postexercise hypotension" OR "post-exercise hypotension" OR PEH OR SBP OR DBP OR MAP),ab,kw  #3 (postexercise OR "post exercise" OR "post-exercise" OR "after exercise" OR "following exercise" OR recovery OR acute),ab,kw  #4 #1 AND #2 AND #3 |
| Scopus | 2026-02-01 | 578 | TITLE-ABS-KEY (("sprint interval training" OR "sprint interval exercise" OR "sprint interval" OR "repeated sprint" OR "repeated-sprint" OR Wingate OR "all-out" OR "supramaximal exercise" OR "supramaximal interval exercise" OR SIT OR SIE) AND ("blood pressure" OR "systolic blood pressure" OR "diastolic blood pressure" OR "mean arterial pressure" OR "postexercise hypotension" OR "post-exercise hypotension" OR PEH OR SBP OR DBP OR MAP) AND (postexercise OR "post exercise" OR "post-exercise" OR "after exercise" OR "following exercise" OR recovery OR acute)) |
| SPORTDiscus | 2026-02-01 | 265 | (TI ("sprint interval training" OR "sprint interval exercise" OR "sprint interval" OR "repeated sprint" OR "repeated-sprint" OR Wingate OR "all-out" OR "supramaximal exercise" OR "supramaximal interval exercise" OR SIT OR SIE) OR AB ("sprint interval training" OR "sprint interval exercise" OR "sprint interval" OR "repeated sprint" OR "repeated-sprint" OR Wingate OR "all-out" OR "supramaximal exercise" OR "supramaximal interval exercise" OR SIT OR SIE)) AND (TI ("blood pressure" OR "systolic blood pressure" OR "diastolic blood pressure" OR "mean arterial pressure" OR "postexercise hypotension" OR "post-exercise hypotension" OR PEH OR SBP OR DBP OR MAP) OR AB ("blood pressure" OR "systolic blood pressure" OR "diastolic blood pressure" OR "mean arterial pressure" OR "postexercise hypotension" OR "post-exercise hypotension" OR PEH OR SBP OR DBP OR MAP)) AND (TI (postexercise OR "post exercise" OR "post-exercise" OR "after exercise" OR "following exercise" OR recovery OR acute) OR AB (postexercise OR "post exercise" OR "post-exercise" OR "after exercise" OR "following exercise" OR recovery OR acute)) |
| Embase | 2026-02-01 | 325 | 1 exp high intensity interval training/  2 exp blood pressure/ or exp ambulatory blood pressure monitoring/  3 ("sprint interval training" or "sprint interval exercise" or "sprint interval" or "repeated sprint" or "repeated-sprint" or Wingate or "all-out" or "supramaximal exercise" or "supramaximal interval exercise" or SIT or SIE).ti,ab.  4 ("blood pressure" or "systolic blood pressure" or "diastolic blood pressure" or "mean arterial pressure" or "postexercise hypotension" or "post-exercise hypotension" or PEH or SBP or DBP or MAP).ti,ab.  5 (postexercise or "post exercise" or "post-exercise" or "after exercise" or "following exercise" or recovery or acute).ti,ab.  6 (1 or 3) and (2 or 4) and 5  7 limit 6 to human |

# Supplementary Table S2. PRISMA flow counts and full-text exclusion reasons.

| **Node** | **Stage** | **Count** | **Interpretation note** |
| --- | --- | --- | --- |
| Database records identified | Identification | 2,300 | Total database hits |
| Duplicate records removed | Identification | 1,425 | Corrected interpretation for arithmetic consistency |
| Records screened | Screening | 875 | 2,300 − 1,425 = 875 |
| Records excluded | Screening | 502 | 875 − 502 = 373 full-text reports |
| Full-text reports assessed | Eligibility | 373 | Full-text eligibility review |
| Full-text reports excluded | Eligibility | 364 | 373 − 364 = 9 included |
| Wrong population | Eligibility reason | 59 | Reported reason total |
| Wrong intervention | Eligibility reason | 216 | Reported reason total |
| Wrong outcomes | Eligibility reason | 62 | Reported reason total |
| Wrong study design | Eligibility reason | 27 | Reported reason total |
| Studies included in qualitative synthesis | Included | 9 | Final included set |
| Studies included in quantitative/descriptive synthesis | Included | 9 | Contributed to at least one synthesis family |

# Supplementary Table S3A. Structured passive-control and 24-h descriptive input rows.

| **Entry ID** | **Study** | **Outcome** | **Time point** | **Comparison** | **n** | **Intervention mean ± SD** | **Comparator mean ± SD** | **Role** |
| --- | --- | --- | --- | --- | --- | --- | --- | --- |
| ANG2015_SBP_2nd_hour | Angadi 2015 | SBP | 2nd hour | SIE vs Control | 11 | 120 ± 11 | 123 ± 8 | SBP earliest >60 min |
| ANG2015_DBP_2nd_hour | Angadi 2015 | DBP | 2nd hour | SIE vs Control | 11 | 67 ± 8 | 70 ± 7 | DBP earliest >60 min |
| ANG2015_SBP_3rd_hour | Angadi 2015 | SBP | 3rd hour | SIE vs Control | 11 | 123 ± 10 | 124 ± 8 | SBP latest >60 min |
| ANG2015_DBP_3rd_hour | Angadi 2015 | DBP | 3rd hour | SIE vs Control | 11 | 70 ± 8 | 70 ± 7 | DBP latest >60 min |
| BUR2012_SBP_90min | Burns 2012 | SBP | 90 min | SIE vs Rest | 10 | 99 ± 10 | 104 ± 10 | SBP earliest/latest >60 min |
| CHA2013_SBP_120min | Chan and Burns 2013 | SBP | 120 min | SIE vs Control | 10 | 109 ± 8 | 117 ± 8 | SBP earliest/latest >60 min |
| CHA2013_DBP_120min | Chan and Burns 2013 | DBP | 120 min | SIE vs Control | 10 | 77 ± 5 | 84 ± 6 | DBP earliest/latest >60 min |
| MCC2023_SBP_24h | McCarthy 2023 | SBP | 24-h average | SIE vs Control | 13 | 121 ± 12 | 123 ± 13 | Single-study descriptive |
| MCC2023_DBP_24h | McCarthy 2023 | DBP | 24-h average | SIE vs Control | 13 | 75 ± 8 | 75 ± 10 | Single-study descriptive |

# Supplementary Table S3B. Protocol-comparator input rows for longer- versus shorter-recovery SIE protocols.

| **Entry ID** | **Study** | **Outcome** | **Time point** | **Comparison** | **n** | **Intervention mean ± SD** | **Comparator mean ± SD** | **Role** |
| --- | --- | --- | --- | --- | --- | --- | --- | --- |
| KET2022_pSBP_45min | Ketelhut 2023b | pSBP | 45 min | 3-min vs 1-min recovery SIE | 30 | 110 ± 8 | 117 ± 11 | pSBP protocol synthesis |
| KET2022_pDBP_45min | Ketelhut 2023b | pDBP | 45 min | 3-min vs 1-min recovery SIE | 30 | 69 ± 8 | 70 ± 9 | pDBP protocol synthesis |
| KET2022_cSBP_45min | Ketelhut 2023b | cSBP | 45 min | 3-min vs 1-min recovery SIE | 30 | 105 ± 8 | 108 ± 11 | Supplementary endpoint |
| KET2023_Older_pSBP_45min | Ketelhut 2023a | pSBP | 45 min | 3-min vs 1-min recovery SIE, older | 12 | 111.5 ± 8 | 119 ± 13 | pSBP protocol synthesis |
| KET2023_Older_pDBP_45min | Ketelhut 2023a | pDBP | 45 min | 3-min vs 1-min recovery SIE, older | 12 | 76 ± 7 | 77 ± 6 | pDBP protocol synthesis |
| KET2023_Older_cSBP_45min | Ketelhut 2023a | cSBP | 45 min | 3-min vs 1-min recovery SIE, older | 12 | 107 ± 7 | 114 ± 12 | Supplementary endpoint |
| KET2023_Young_pSBP_45min | Ketelhut 2023a | pSBP | 45 min | 3-min vs 1-min recovery SIE, young | 12 | 110 ± 10 | 115 ± 11 | pSBP protocol synthesis |
| KET2023_Young_pDBP_45min | Ketelhut 2023a | pDBP | 45 min | 3-min vs 1-min recovery SIE, young | 12 | 67 ± 10 | 66 ± 9 | pDBP protocol synthesis |
| KET2023_Young_cSBP_45min | Ketelhut 2023a | cSBP | 45 min | 3-min vs 1-min recovery SIE, young | 12 | 104 ± 9 | 105 ± 11 | Supplementary endpoint |

# Supplementary Table S4. Cochrane RoB 2 domain judgments for included studies.

| **Study** | **Randomization** | **Carryover / period effects** | **Deviations** | **Missing data** | **Measurement** | **Selective reporting** | **Overall** |
| --- | --- | --- | --- | --- | --- | --- | --- |
| Stuckey 2012 | Low | Low | Low | Low | Low | Some concerns | Some concerns |
| Angadi 2015 | Some concerns | Low | Low | High | Low | Some concerns | High |
| Burns 2012 | Some concerns | Some concerns | Low | Low | Low | Some concerns | Some concerns |
| Graham 2016 | Some concerns | Low | Some concerns | Some concerns | Low | Some concerns | Some concerns |
| Chan and Burns 2013 | Some concerns | Low | Low | Low | Low | Some concerns | Some concerns |
| Jones 2021 | Some concerns | Low | Low | Low | Low | Some concerns | Some concerns |
| Ketelhut 2023b | Some concerns | Low | Low | Low | Low | Some concerns | Some concerns |
| Ketelhut 2023a | Some concerns | Low | Low | Low | Low | Some concerns | Some concerns |
| McCarthy 2023 | Low | Some concerns | Low | Some concerns | Low | Some concerns | Some concerns |

# Supplementary Table S5. Sensitivity analyses across assumed within-participant correlations.

| **Outcome** | **Variant** | **Assumed r** | **k** | **Exploratory summary MD** | **95% CI low** | **95% CI high** | **I² (%)** |
| --- | --- | --- | --- | --- | --- | --- | --- |
| SBP | Earliest >60 min | 0.00 | 3 | −5.62 | −10.14 | −1.09 | 0 |
| SBP | Earliest >60 min | 0.30 | 3 | −5.63 | −9.43 | −1.83 | 0 |
| SBP | Earliest >60 min | 0.50 | 3 | −5.65 | −8.88 | −2.43 | 0 |
| SBP | Earliest >60 min | 0.70 | 3 | −5.59 | −8.57 | −2.61 | 27.45 |
| SBP | Earliest >60 min | 0.90 | 3 | −5.48 | −8.42 | −2.53 | 72.71 |
| SBP | Latest >60 min | 0.00 | 3 | −4.83 | −9.26 | −0.39 | 0 |
| SBP | Latest >60 min | 0.30 | 3 | −4.80 | −8.98 | −0.61 | 20.46 |
| SBP | Latest >60 min | 0.50 | 3 | −4.76 | −8.96 | −0.57 | 42.75 |
| SBP | Latest >60 min | 0.70 | 3 | −4.73 | −8.92 | −0.55 | 65.05 |
| SBP | Latest >60 min | 0.90 | 3 | −4.71 | −8.83 | −0.59 | 87.36 |
| DBP | Earliest >60 min | 0.00 | 2 | −5.51 | −9.34 | −1.68 | 0 |
| DBP | Earliest >60 min | 0.30 | 2 | −5.36 | −9.22 | −1.51 | 28.01 |
| DBP | Earliest >60 min | 0.50 | 2 | −5.26 | −9.15 | −1.37 | 48.24 |
| DBP | Earliest >60 min | 0.70 | 2 | −5.16 | −9.06 | −1.25 | 68.47 |
| DBP | Earliest >60 min | 0.90 | 2 | −5.05 | −8.97 | −1.13 | 88.69 |
| DBP | Latest >60 min | 0.00 | 2 | −3.80 | −10.63 | 3.04 | 66.59 |
| DBP | Latest >60 min | 0.30 | 2 | −3.71 | −10.56 | 3.14 | 76.49 |
| DBP | Latest >60 min | 0.50 | 2 | −3.65 | −10.50 | 3.21 | 83.10 |
| DBP | Latest >60 min | 0.70 | 2 | −3.59 | −10.45 | 3.27 | 89.70 |
| DBP | Latest >60 min | 0.90 | 2 | −3.53 | −10.39 | 3.33 | 96.31 |

# Supplementary Table S6. GRADE evidence profile.

| **Evidence family** | **Outcome** | **Studies / participants** | **Downgrades** | **Final certainty** | **Summary statement** |
| --- | --- | --- | --- | --- | --- |
| Primary passive-control structured/exploratory summary | SBP, earliest >60 min | 3 crossover studies; 31 participants | Risk of bias; imprecision | Low | Exploratory summary MD −5.65 mmHg (95% CI −8.88 to −2.43); I² = 0% |
| Primary passive-control structured/exploratory summary | DBP, earliest >60 min | 2 crossover studies; 21 participants | Risk of bias; inconsistency; imprecision | Very low | Exploratory summary MD −5.26 mmHg (95% CI −9.15 to −1.37); I² = 48% |
| Protocol-comparator structured/exploratory summary | Peripheral SBP at 45 min, longer vs shorter recovery | 3 crossover estimates; 54 participants | Indirectness; reporting limitations | Low | Exploratory summary MD −6.67 mmHg (95% CI −9.41 to −3.92); I² = 0% |
| Protocol-comparator structured/exploratory summary | Peripheral DBP at 45 min, longer vs shorter recovery | 3 crossover estimates; 54 participants | Indirectness; imprecision | Low | Exploratory summary MD −0.68 mmHg (95% CI −2.84 to 1.48); I² = 0% |
| Descriptive exact single-study evidence | 24-h ambulatory SBP/DBP after SIE vs control | 1 study; 13 participants with valid ABP | Single-study evidence; no pooling | Very low | SBP 121 ± 12 vs 123 ± 13 mmHg; DBP 75 ± 8 vs 75 ± 10 mmHg |

# Supplementary Table S7. Review-level contextual safeguards.

| **Item** | **Procedure** | **Role in current review** |
| --- | --- | --- |
| AMSTAR-2 | Applied to relevant review-level sources used for background and contextualization | Interpretive transparency only; not used to rate primary trials |
| Corrected covered area | Considered overlap among review-level evidence when contextualizing previous reviews | Prevented double counting of review-level conclusions |
| Primary-study synthesis | Only individual acute experimental studies contributed quantitative effect sizes | Avoided combining published review estimates with primary data |

# Supplementary Figure S1. Overall risk-of-bias summary plot across the included studies.


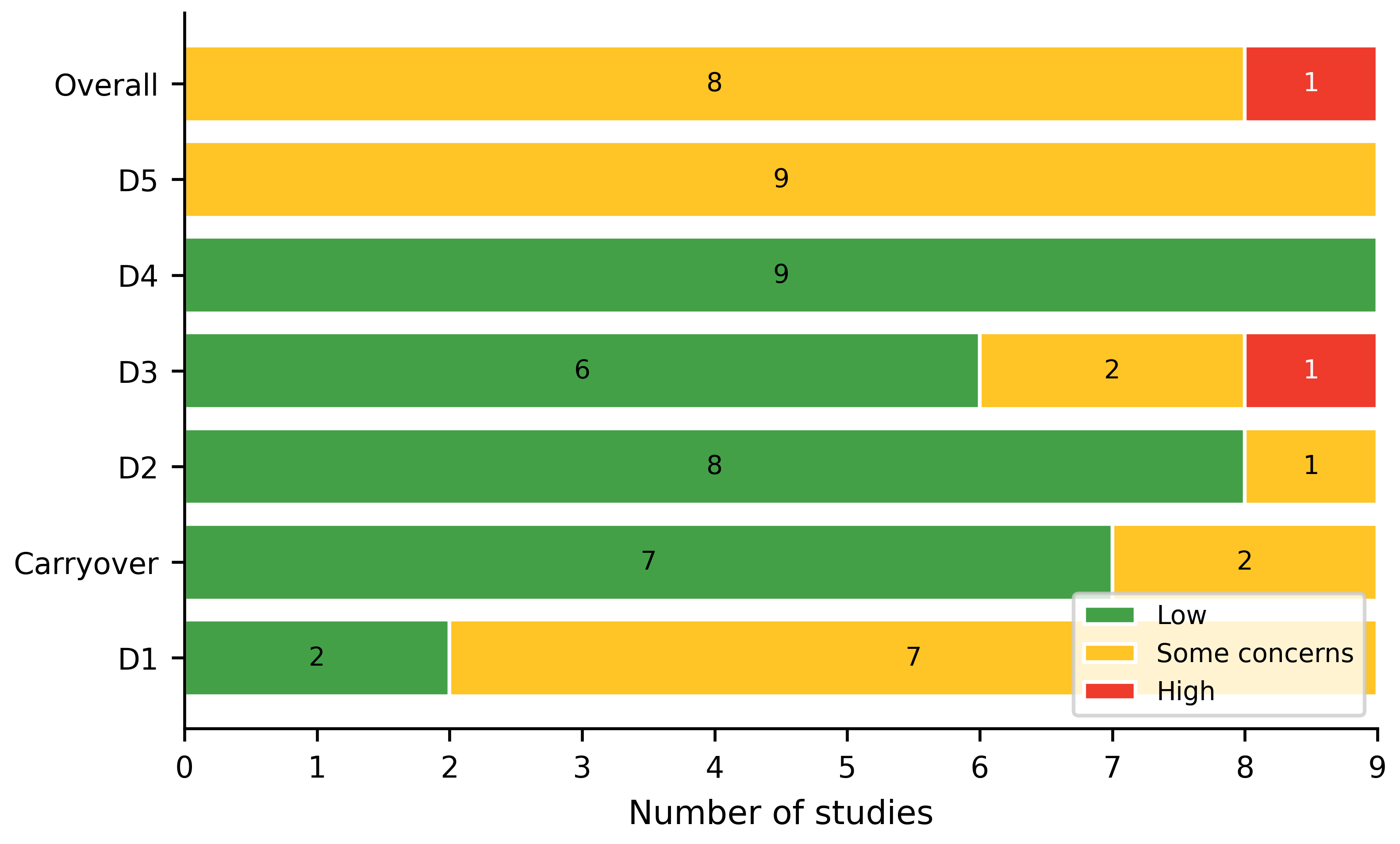


# Supplementary Figure S2. Exploratory forest plot of peripheral diastolic blood pressure at 45 min comparing longer versus shorter recovery intervals between sprint bouts. Negative values favor the longer-recovery protocol.


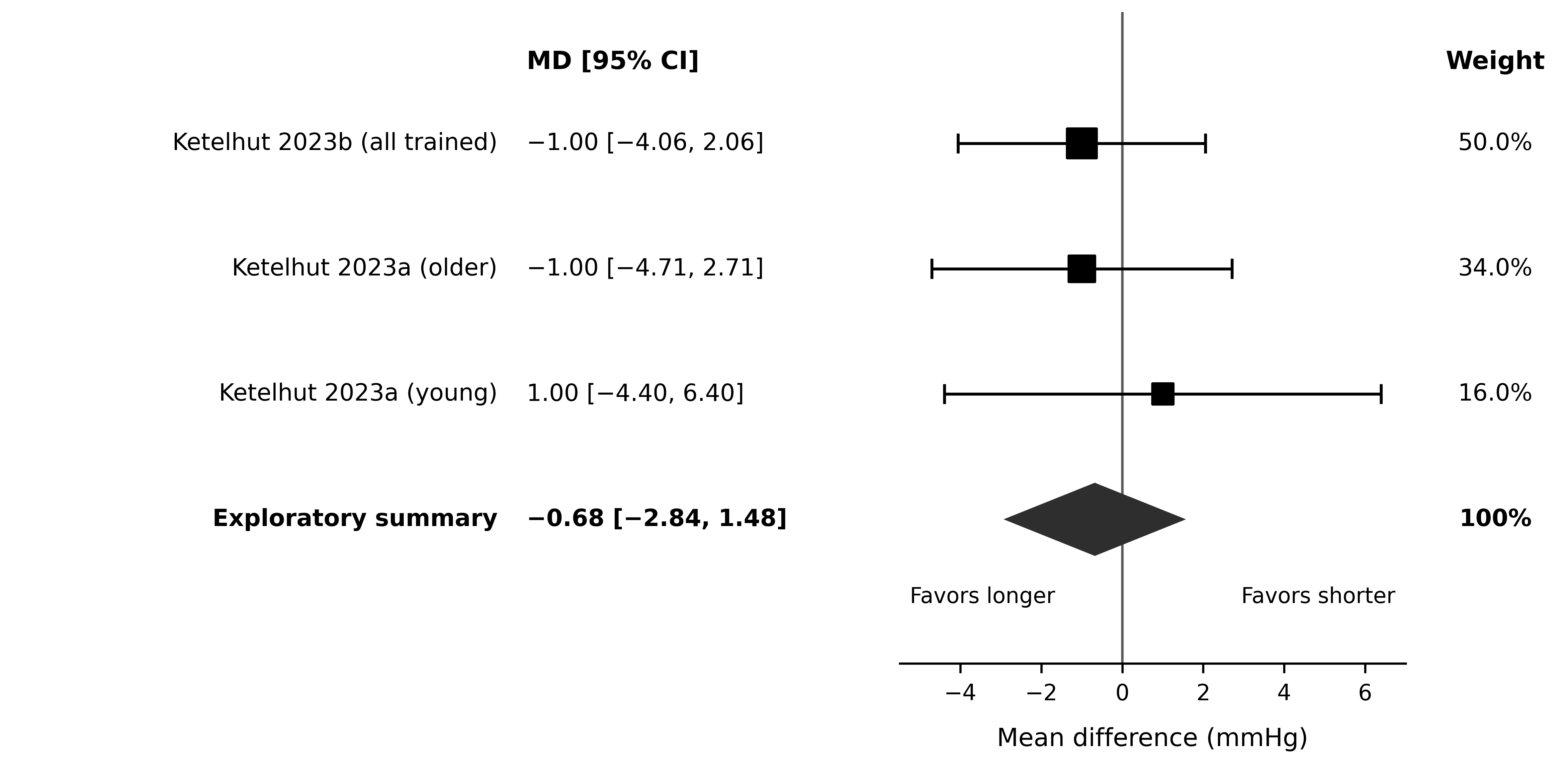


# Supplementary Figure S3. Exploratory funnel plot for the primary passive-control SBP family. Because only three studies contributed to this synthesis, the plot is shown for completeness only and should not be interpreted as a formal test of small-study effects.


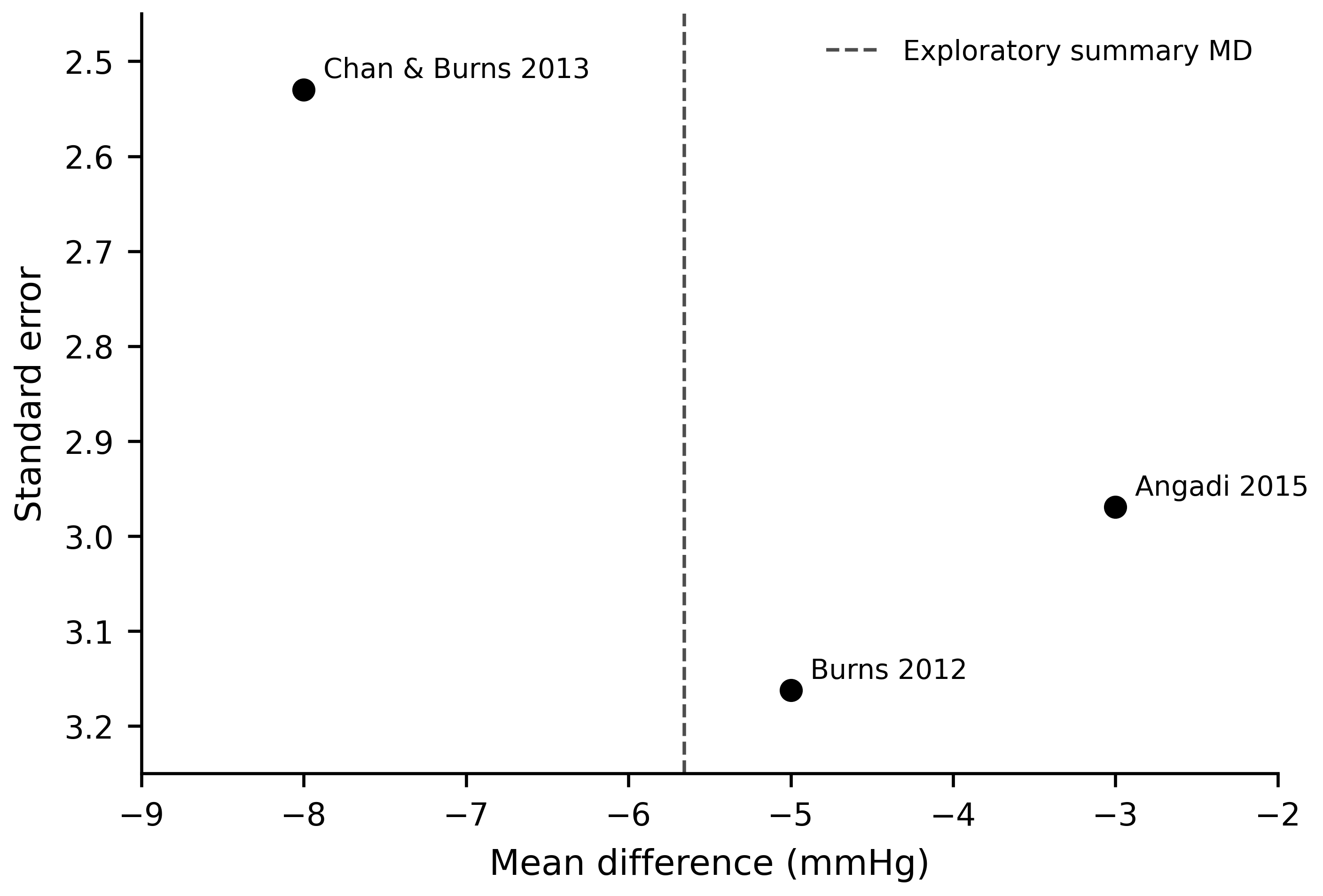


# Supplementary Figure S4. Evidence map summarizing study families, comparator classes, outcome domains and extractability for structured synthesis and exploratory quantitative summaries.


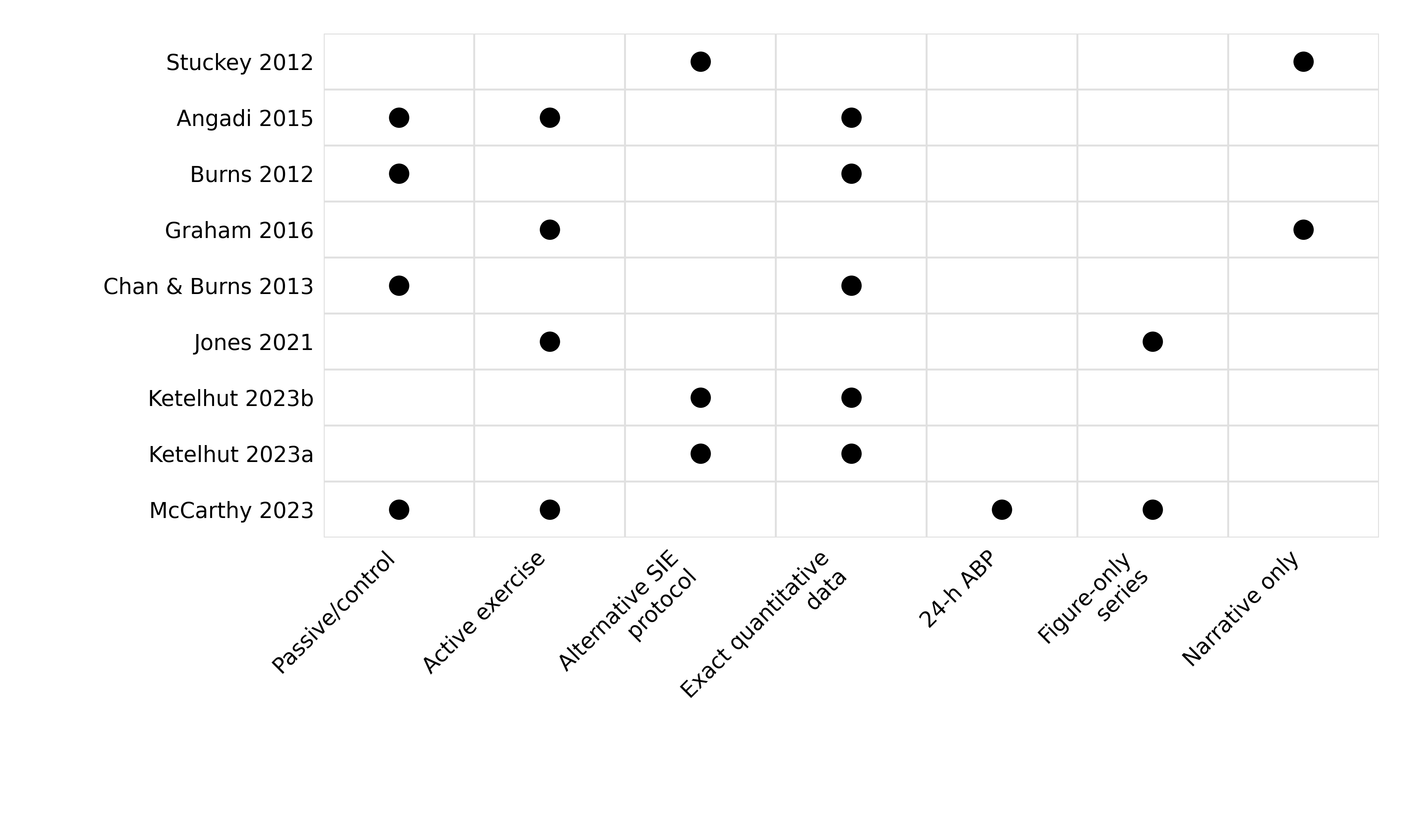


# Supplementary Figure S5. Exploratory leave-one-out sensitivity analysis for the primary passive-control SBP synthesis. Negative summary values favor SIE.


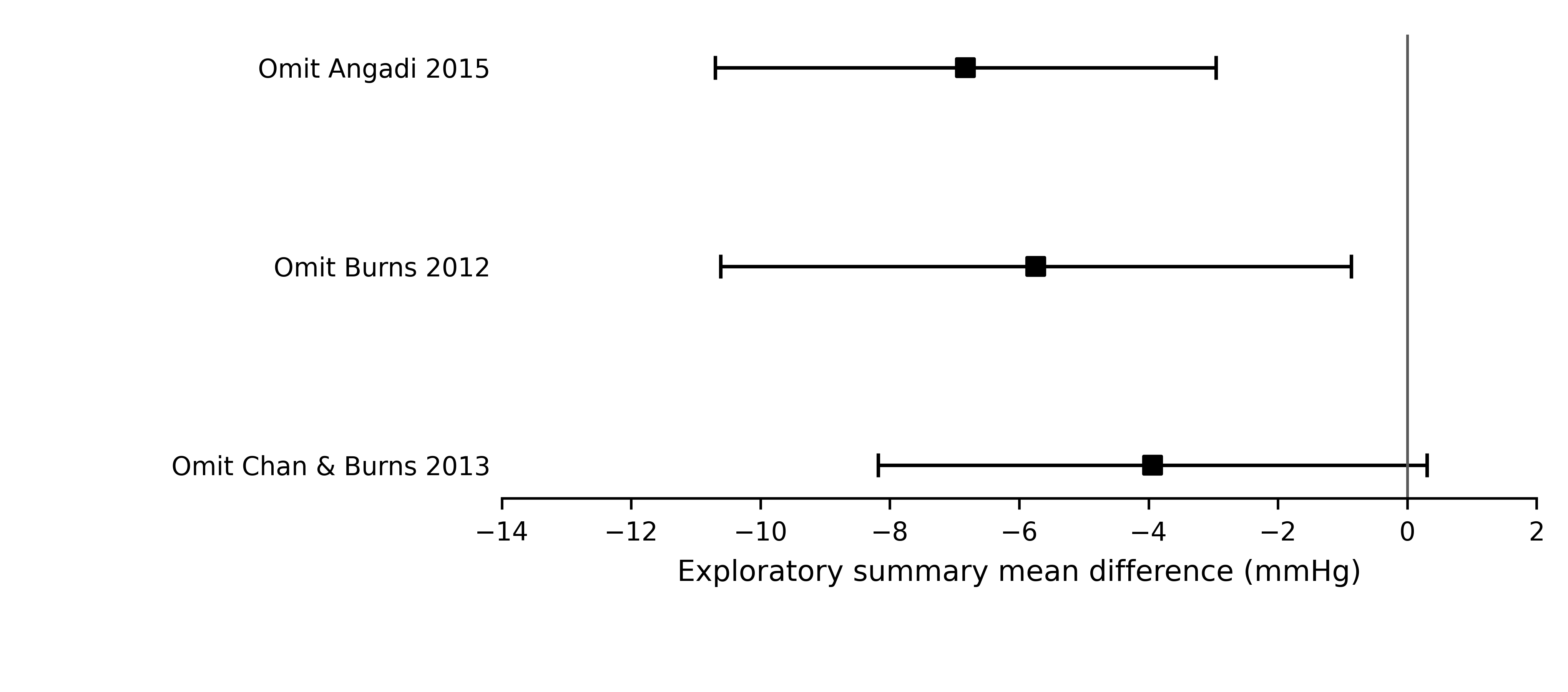

Supplement: Supplementary file 1 [file SupplementaryFile1.docx]
