# Supplementary material for: Acute post-exercise blood pressure responses to sprint interval exercise in humans: a systematic review and structured synthesis
Source: Front Physiol. 2026 Jul 8;17:1865794. doi: 10.3389/fphys.2026.1865794 (PMC13388036; doi:10.3389/fphys.2026.1865794)
Supplement: Supplementary file 3 [file SupplementaryFile3.docx]

# Supplementary File 3. Reproducibility code for the SIE acute post-exercise BP systematic review

# Purpose: reproduce crossover-aware effect sizes, assumed-correlation sensitivity analyses,

# and exploratory DerSimonian-Laird random-effects summaries reported in the revised manuscript.

# Requirements: R >= 4.3.0 and metafor package. Install with install.packages("metafor") if needed.

suppressPackageStartupMessages(library(metafor))

paired_se <- function(sd_i, sd_c, n, r = 0.50) {

sqrt((sd_i^2 + sd_c^2 - 2 * r * sd_i * sd_c) / n)

}

run_summary <- function(dat, r = 0.50, label = "analysis") {

dat$md <- dat$mean_i - dat$mean_c

dat$sei <- paired_se(dat$sd_i, dat$sd_c, dat$n, r)

fit <- rma.uni(yi = dat$md, sei = dat$sei, method = "DL", test = "z")

out <- data.frame(

label = label,

r = r,

k = fit$k,

MD = as.numeric(fit$b),

CI_low = fit$ci.lb,

CI_high = fit$ci.ub,

I2 = fit$I2,

tau2 = fit$tau2

)

list(data = dat, model = fit, summary = out)

}

primary_sbp_earliest <- data.frame(

study = c("Angadi 2015 (2nd hour)", "Burns 2012 (90 min)", "Chan & Burns 2013 (120 min)"),

n = c(11, 10, 10),

mean_i = c(120, 99, 109), sd_i = c(11, 10, 8),

mean_c = c(123, 104, 117), sd_c = c(8, 10, 8)

)

primary_dbp_earliest <- data.frame(

study = c("Angadi 2015 (2nd hour)", "Chan & Burns 2013 (120 min)"),

n = c(11, 10),

mean_i = c(67, 77), sd_i = c(8, 5),

mean_c = c(70, 84), sd_c = c(7, 6)

)

primary_sbp_latest <- data.frame(

study = c("Angadi 2015 (3rd hour)", "Burns 2012 (90 min)", "Chan & Burns 2013 (120 min)"),

n = c(11, 10, 10),

mean_i = c(123, 99, 109), sd_i = c(10, 10, 8),

mean_c = c(124, 104, 117), sd_c = c(8, 10, 8)

)

primary_dbp_latest <- data.frame(

study = c("Angadi 2015 (3rd hour)", "Chan & Burns 2013 (120 min)"),

n = c(11, 10),

mean_i = c(70, 77), sd_i = c(8, 5),

mean_c = c(70, 84), sd_c = c(7, 6)

)

protocol_psbp <- data.frame(

study = c("Ketelhut 2023b (all trained)", "Ketelhut 2023a (older)", "Ketelhut 2023a (young)"),

n = c(30, 12, 12),

mean_i = c(110, 111.5, 110), sd_i = c(8, 8, 10),

mean_c = c(117, 119, 115), sd_c = c(11, 13, 11)

)

protocol_pdbp <- data.frame(

study = c("Ketelhut 2023b (all trained)", "Ketelhut 2023a (older)", "Ketelhut 2023a (young)"),

n = c(30, 12, 12),

mean_i = c(69, 76, 67), sd_i = c(8, 7, 10),

mean_c = c(70, 77, 66), sd_c = c(9, 6, 9)

)

analyses <- list(

run_summary(primary_sbp_earliest, r = 0.50, label = "SBP earliest >60 min"),

run_summary(primary_dbp_earliest, r = 0.50, label = "DBP earliest >60 min"),

run_summary(primary_sbp_latest, r = 0.50, label = "SBP latest >60 min"),

run_summary(primary_dbp_latest, r = 0.50, label = "DBP latest >60 min"),

run_summary(protocol_psbp, r = 0.50, label = "Protocol pSBP 45 min"),

run_summary(protocol_pdbp, r = 0.50, label = "Protocol pDBP 45 min")

)

main_summary <- do.call(rbind, lapply(analyses, `[[`, "summary"))

print(main_summary)

r_values <- c(0, 0.3, 0.5, 0.7, 0.9)

sensitivity <- do.call(rbind, lapply(r_values, function(rr) {

rbind(

run_summary(primary_sbp_earliest, rr, "SBP earliest >60 min")$summary,

run_summary(primary_sbp_latest, rr, "SBP latest >60 min")$summary,

run_summary(primary_dbp_earliest, rr, "DBP earliest >60 min")$summary,

run_summary(primary_dbp_latest, rr, "DBP latest >60 min")$summary

)

}))

print(sensitivity)

# Optional exports to the working directory:

write.csv(main_summary, "reproduced_main_summaries.csv", row.names = FALSE)

write.csv(sensitivity, "reproduced_sensitivity_correlation_sweep.csv", row.names = FALSE)

cat("\nmetafor package version:\n")

print(utils::packageVersion("metafor"))

cat("\nR session information:\n")

sessionInfo()
